# Dose transition pathways: a design, analysis and operational tool for dose-finding trials using model-based designs

**DOI:** 10.1186/1745-6215-16-S2-O13

**Published:** 2015-11-16

**Authors:** Christina Yap, Lucinda Billingham, Charles Craddock, John O'Quigley

**Affiliations:** 1 Cancer Research UK Clinical Trials Unit, University of Birmingham, Birmingham, UK; 2 Centre for Clinical Haematology, Queen Elizabeth Hospital, Birmingham, UK; 3 Université Paris VI, Paris, France

## 

Despite the mounting evidence for the superior performance of model-based dose-finding designs in identifying the maximum tolerated dose compared to traditional up-and-down designs, its use is still very limited in medical literature as there remain several challenges in implementation.

We introduce the use of the Dose Transition Pathways, and demonstrate how it can be used to overcome several commonly faced challenges, including: Firstly, the challenge of communicating to clinicians about how the design works and the rationale for using a method with substantially higher level of complexity. Secondly, operational challenges including trialists' impressions that dose recommendations come from a “black-box”, which contrast unfavourably with the transparent simple rules of a traditional 3+3 design. Possible delays may thus result, particularly for each dose recommendation. Thirdly, methodological challenges include how to choose an appropriate bespoke adaptive design that is applicable in practice.

We will illustrate how the Dose-Transition Pathway can be used to aid in planning and designing an adaptive model-based dose-finding trial, specifically tailored to its requirements, with a case study of a Phase I Acute Myeloid Leukaemia and Myelodysplastic Syndrome trial. By aiding trialist's understanding of the working of such adaptive designs in practice, as well as achieving a seamless, clear transition for each dose-recommendation, the Dose Transition Pathways will serve as a valuable tool in the implementation of adaptive model-based designs in dose-finding trials.

